# Clinical characteristics of vulnerable populations hospitalized and diagnosed with COVID-19 in Buenos Aires, Argentina

**DOI:** 10.1038/s41598-021-87552-w

**Published:** 2021-05-06

**Authors:** A. Yacobitti, L. Otero, V. Doldan Arrubarrena, J. Arano, S. Lage, M. Silberman, M. Zubieta, I. Erbetta, P. Danei, G. Baeck, V. Vallejos, F. Cavalli, N. Calderón, M. Di Gregorio, V. Hernandez, D. Bruno, B. Rodera, I. Macherett, M. Parisi, M. Gallastegui, A. Paz, R. Bernardi, S. Azcárate, A. Hraste, I. Caridi, L. Boechi, P. Salgado, S. Kochen

**Affiliations:** 1 Network Patient Management, Hospital El Cruce N Kirchner, F. Varela, Pcia Buenos Aires Argentina; 2 Planning Area, Hospital El Cruce N Kirchner, F. Varela, Pcia Buenos Aires Argentina; 3 General Ward, Hospital El Cruce N Kirchner, F. Varela, Pcia Buenos Aires Argentina; 4 Intensive Therapy Unit, Hospital El Cruce N Kirchner, F. Varela, Pcia Buenos Aires Argentina; 5 Laboratory, Hospital El Cruce, F. Varela, Pcia Buenos Aires Argentina; 6 Administrative Area, Hospital Module N° 11, F. Varela, Pcia Buenos Aires Argentina; 7 Administrative Area, UPA N° 11, F. Varela, Pcia Buenos Aires Argentina; 8 Patient Admission, Hospital Mi Pueblo, F. Varela, Pcia Buenos Aires, Argentina; 9 Prompt Attention Unit, UPA N° 5, A. Brown, Pcia Buenos Aires Argentina; 10 Administrative Area, UPA N° 5 and Module N° 9, A. Brown, Pcia Buenos Aires Argentina; 11 Administrative Area, Hospital L. Meléndez, A. Brown, Pcia Buenos Aires Argentina; 12 Statistics, Hospital Oñativia, A. Brown, Pcia Buenos Aires Argentina; 13 Administrative Area, Hospital Oñativia, A. Brown, Pcia Buenos Aires Argentina; 14 Medical Clinic, Iriarte Hospital, Quilmes, Pcia Buenos Aires Argentina; 15 Intensive Therapy Unit, Iriarte Hospital, Quilmes, Pcia Buenos Aires Argentina; 16 Oller HospitalOller Hospital, Quilmes, Pcia Buenos Aires Argentina; 17 Administrative Area, UPA N° 17, Quilmes, Pcia Buenos Aires Argentina; 18 Intensive Therapy Unit, Evita Pueblo Hospital, Berazategui, Pcia Buenos Aires Argentina; 19 Patient Management, Evita Pueblo Hospital, Berazategui, Pcia Buenos Aires Argentina; 20 Institute of Calculation, FCEN, UBA and CONICET, Ciudad de Buenos Aires, Argentina; 21 Public Health Research Institute, University of Buenos Aires, Caba, Argentina; 22 Neurosciences and Complex Systems Unit (EnyS), CONICET- Hosp. El Cruce “N. Kirchner” - Univ. National A. Jauretche, Fac. Med, Univ. Buenos Aires, Av Calchaqui 5401, CP B1888AAE F. Varela, Province Buenos Aires Argentina

**Keywords:** Diseases, Infectious diseases, Viral infection

## Abstract

There is not in Argentina publications regarding the presentation of patients with COVID-19 requiring hospitalized and emergency care in vulnerable populations (lower incomes and less education tend at greater risk for poor health status and healthcare access), and it has few reports in developing countries. The objective is to determine whether in the care of vulnerable patients, to succeed against COVID-19, multiple public health tools and interventions will be needed to minimize morbidity and mortality. The study is a prospective cohort investigation of patients with lab-confirmed COVID-19, who required to any of the Health Centers response from April 8, 2020, to August 18, 2020. In Buenos Aires Metropolitan Area (AMBA), April 8, 2020 the virus was identified in patients hospitalized in the "Southeast Network" (SN), AMBA. SN covering an area of 661 square kilometers, with 1.8 million inhabitants residing in urban, and rural areas. A total of 14 health centers with different levels of care complexity provide care to patients in the region. The information of each patient with COVID-19 evaluated by SN, was incorporated in an Epidemiological Dashboard. The investigation was designed and reported with consideration of observational studies in epidemiology. We describe the hospitals presentation and care of persons who required SN response and were ultimately diagnosed with COVID-19. From April 8, 2020, to August 18, 2020, were included 1495 patients with lab-confirmed COVID-19 in SN. A total of 58% patients were men, and the mean age (SD) was 48.9 (15.59) years. Eighty one percent patients with pre-existing diseases, most frequent hypertension and diabetes, but hypertension, chronic lung disease, and cardiovascular disease presented higher risk. A total of 13% were hospitalized in Intensive Therapy Unit. The mortality of the cohort was 9.77%. Mortality was higher for patients aged 65 or more (OR 5.09), and for those had some pre-existing disease (OR 2.61). Our observations are consistent with reports demonstrating older persons, and those with comorbidities have the highest risk of mortality related to COVID-19. However, unlike other reports from developed or some developing countries, the mortality in our study is lower. This finding may be related to age of our cohort is younger than other published. Also, the health system was able to respond to the demand.

## Introduction

The coronavirus disease 2019 (COVID-19) pandemic was first reported in Hubei Province, China, in December 2019^1^. The initial Argentina case of COVID-19 was reported on March 3, 2020^2^, in Buenos Aires Metropolitan Area (AMBA)^3^. In April 8, 2020 the virus was identified in patients hospitalized in the "Southeast Network", AMBA. The Hospital "El Cruce, Néstor Kirchner" (HEC), following the modality of network implemented since its creation in 2007, organized a network system with the Health Centers of the region especially for the pandemic, named "South East Network "^4^. Subsequently, lab-confirmed cases of COVID-19 increased exponentially in AMBA area, also but lesser extent, in other parts of Argentina.

Although the clinical profile of patients has been reported^5–7^, no publications in Argentina regarding the presentation of patients with COVID-19 requiring hospitalized and emergency care in vulnerable populations.

In developing countries, especially in the care of vulnerable patients, to succeed against COVID-19, multiple public health tools and interventions will be needed to minimize morbidity and mortality related to COVID19. Although extreme public health interventions, for example, lockdowns and stay-at-home mandates, were initially critical to flattening the curve. Since the beginning of the pandemic in light of the situation reported in countries that have begun the pandemic before in Argentina^8–10^, it decide to create a Southeast Network in order to remedy the inefficiencies in the management of information and coordination between the different public (unpaid) health institutions in the region, and increase the number of available hospital beds and human resources.

In this study, we describe the hospitals presentation and care of persons who required Southeast Network (SN) response and were ultimately diagnosed with COVID-19 to offer actionable understandings to help to inform best practice.

## Methods

### Study design, setting, and population

The study is a prospective cohort investigation of patients with lab-confirmed COVID-19 in SN, who required to any of the Centers response from April 8, 2020, to August 18, 2020. The recruitment was consecutive.

We used a flow chart, following the STROBE guidelines^11^. The evaluation of the patient begins with the triage in emergency room, where the patient is classified as a suspected case of COVID-19. Swabbing for PCR determination of SARS-CoV-2 is performed.

While waiting for the PCR result, the patient is considered to be infected with SARS-CoV-2 and all precautions for personal protection and patient isolation are taken.

The clinical, laboratory and radiological evaluation is carried out.

The severity of the clinical picture is classified to determine the place of hospitalization and indicate the treatment.

The inclusion criteria were hospitalized patients, in the first stage of the study, patients with suspected and confirmed COVID were admitted. But in the data analysis, only patients with a confirmed diagnosis of COVID-19 by PCR determination of SARS-CoV-2 in nasopharyngeal exudate were included. Excluded criteria was patients without a confirmed diagnosis of COVID-19 and not hospitalized.

The investigation was designed and reported with consideration of observational studies in epidemiology reporting guideline of Health Ministry of Province of Buenos Aires (http://www.ms.gba.gov.ar/ssps/investigacion/DocTecnicos/TipoInvestigaciones-Identificacionriesgos.pdf. Accessibility verified August 24, 2020.) ^12^. Because the investigation was considered minimal risk, the requirement for consent was only verbal to patients or their relatives. (Ethics Committee, El Cruce Hospital N Kirchner).

All experimental protocols of the study were approved by the ethics committee of 'Hospital El Cruce', Argentina.

COVID-19 was diagnosed by real-time reverse transcription–polymerase chain reaction (RT-PCR) detection of SARS-CoV-2 from nasopharyngeal swabs. Molecular techniques based RT-PCR are considered the gold standard for COVID-19 diagnosis, real-time RT-PCR detects amplified SARS-CoV-2 genome^13^.

The RT-PCR determination was centralized in two Laboratories (El Cruce Hospital, and National Quilmes University), it has been common to all patients. Test results were available a median 1–3 days after the SN encounter.

SN is a large periurban region, covering an area of 661 square kilometers, with 1.8 million inhabitants residing in urban, suburban, and rural areas. Argentina is considered a developing country with a high human development index (0.830 in 2018), however there are significant structural development gaps and heterogeneities between different areas. The municipalities of the southeast region are located in the lowest ranking of the municipalities of the Province of Buenos Aires^4^. A total of 14 health centers with different levels of care complexity provide care to patients in the region. The Centers are administered by the Ministry of Health of the Nation, by Province of Buenos Aires, of both, and by Municipalities (Supplementary appendix).

The SN medical response is 2 tiered. The first tier is provided by medical doctors (MD) in Emergency Room of each Health Center, all patients go to the emergency room, and majority consult spontaneously in the Center of their neighborhoods.

If the patients had symptoms compatibles con COVID-19 and more severe illness, it was decided whether they should be hospitalized as suspected. The second-tier response in confirmed cases comprises also MD in Intensive Therapy Unit (ITU) stay, or General Ward with oxygen therapy (GWo), or General Ward (GW) stay.

### Ethical approval

The investigation was designed and reported with consideration of observational studies in epidemiology reporting guideline of Health Ministry of Province of Buenos Aires. Because the investigation was considered minimal risk, the requirement for consent was only verbal to patients or their relatives. All experimental protocols of the study were approved by the ethics committee of 'Hospital El Cruce', Argentina.

### Consent for publication

I confirm because the investigation was considered minimal risk, the requirement for consent was only verbal to patients or their relatives. (Ethics Committee, El Cruce Hospital N Kirchner).

## Data sources

The information of each patient with COVID-19 evaluated by SN, was incorporated in an Epidemiological Dashboard, created by specialists (CI, BL) from the design of software especially for this project. The current investigation used from April 8, the electronic medical record incorporated the diagnosis of COVID-19, suspected or known. In a first stage, a "window" was established that allowed the visualization of the occupation and availability of beds by establishment and by sector. In the Dashboard each patient had an identification (ID) generated automatically, name, identity document and address, gender, age, date of admission are incorporated, the system automatically generates the days of hospitalization. Clinical risk was adapted from the National Health Ministry guidelines^14^, in three categories. Mild, when patients have unilateral radiological involvement and SatO_2_ is > 95%, the Internment in WGo, or WG is decided.

Moderate, when radiological involvement is bilateral, patients saturate below 95%, they are admitted to WGo. Severe, when they present ATS/IDSA (American Thoracic Society-ATS) (Infectious Diseases Society of America -IDSA-) criteria: one of two major (-Need for invasive MRA- Septic shock) or three minor (-Tachypnea ≥ 30/min.- PaO_2_/FiO_2_ < 250, Confusion/disorientation Multilobar infiltrates, Urea> 42 mg/100 ml, Leukopenia (<4000/mm^3^), Plaquetopenia (< 100,000/mm^3^), Temperature < 36 °C, Hypotension requiring aggressive hydration). They are admitted to ITU. The safe discharge for the patient and for third parties, was used for those patients who in case of being discharged from hospital, could not return home, because they did not have or because they lived in crowded conditions, and could not be isolated. A socio-economic and food security survey was started, but its analysis was not yet completed, for this reason it was not included in this study.

As the Dashboard and the software is a new instrument, a help desk was implemented on the use of the software. From the beginning a monitoring and follow-up of the data entry was organized twice a day, morning and night, by the HEC technical team (DAV, OL, YA). A "Procedures Manual" was prepared and distributed among the users of the Project.

### Statistical analysis

We analyzed the distribution of the characteristics of the entire hospitalized population and it was stratified by type of bed (ITU, WGo, WG). The characteristics in relation to clinical and safe discharge were compared, we used descriptive statistics, χ^2^ and Fisher's exact test for categorical variables, and t and Wilcoxon's test for continuous variables. Also, to analyze risk factors, we used multivariate analysis and logistic regression. All analyzes were performed using SPSS version 24 statistical software (IBM Corp). A *P* ≤ 0.05 was considered statistically significant, all the tests were 2- tailed.

## Results

From April 8, 2020, to August 18, 2020, 1,495 patients with lab-confirmed COVID-19 in SN were included.

**Figure 1:**
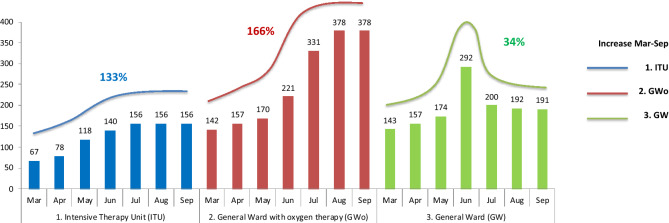
Availability of beds in Southeast Network since March 2020 to August 2020. Availability of beds by area, in Intensive Therapy Unit (ITU) stay, or General Ward with oxygen therapy (GWo), or General Ward (GW) stay. And percentage of increase in beds from March to September.

At the start of pandemic, March 2020, the SN had 67 beds in ITU, and now 156 beds are available, an increase of 133% (Fig. 1). It also increased in GWo area from 142 to 378 beds, an expansion of 166% (Fig. 1), and in GW area from 143 to 191 beds, an increase of 34%.

We did not have extra-hospital isolation beds in the NS. This space began to be incorporated for the first time to the Health Service from the need to have places that allow to have patients with suspected or diagnosed COVID-19 that did not require medical care, but that had to be isolated to avoid contagion to their contacts, and that they could not stay in their homes, because they did not have house or because they did not meet the appropriate conditions for isolation. We have 883 extra-hospital beds (208 in Quilmes, 200 in Almirante Brown, 200 in Florencio Varela and 275 in Berazategui).

The percentage of occupancy of beds on August 18, was for ICU, patients with COVID-19 50% and for suspected patients 13%. In GWo 38% COVID-19 patients and 27% suspected patients, and for GW 21% COVID-19 patients and 20% suspected patients.

This cohort presented an increase exponential since June 2020, similar to that observed in AMBA, in April 8 we had 15 patients hospitalized, to August 18 there was a total of 235 patients hospitalized.

Of the 1495 patients, 690 (46%) cases were discharged to their home, 140 (9%) subjects were discharged extra-hospital isolation, 155 (10%) individuals were transferred out of southeast network, and 364 (24%) patients remain hospitalized (Table 1).

**Table 1:** Follow up of Patients Hospitalized With Coronavirus Disease in Health Centers of Southeast Network.

Follow up of patients hospitalized	No. (%)
Discharge Home	690 (46)
Discharge Extra-hospital isolation	140 (9)
Transfer out of Southeast network (SN)	155 (10)
Remain Hospitalized	364 (24)
Deaths	146 (10)
Total	1495

A total of 826/1429 (58%) patients were men, and the mean age (SD) was 48.9 (15.59) years (Table 2). Of total population studied, we were able to obtain information on their health history in 1249 (84%) patients, 1.012 (81%) patients with chronic comorbidities. A total of 191 patients (13%) were hospitalized in ITU (Table 2), of this group 121 (63%) presented serious clinical risk, 170 (89%) subjects had at least one pre-existing disease, most frequent were hypertension and diabetes, and 77 (42%) patients had , it was unknown for 8 patients. In GWo, 1109 (74%) patients were admitted, with serious clinical risk 60 (5%) subjects, 752 (80%) patients with pre-existing disease, the most frequent were obesity, diabetes and hypertension, whereas health history was unknown for 166 (15%) patients; and 461 (44%) patients had safe discharge, it was unknown for 58 patients (5%). And in GW, 195 (13%) patients were admitted, with serious clinical risk 6 (3%) subjects, 90 (73%) patients with pre-existing disease, the most frequent were diabetes and hypertension and chronic pulmonary disease, it was unknown for 72 (37%) patients; and 116 (65%) cases presented safe discharge, it was unknown for 16 (8%) patients (Table 2).

**Table 2:** Characteristics of Patients Hospitalized with Coronavirus Disease 2019 in Health Centers of Southeast Network.

Characteristics	No. (%)
Male	826 (58)
Women	603 (42)
Age, mean (SD)	48.9 (15.59)
Pre-existent diseases^1^	1012 (81)
^1^Pre-existent diseases	No. (%)
Hypertension	259 (21)
Mellitus diabetes	187 (15)
Chronic lung disease	101 (8)
Cardiovascular disease	92 (7)
Severe obesity	90 (7)
Chronic kidney disease	31 (3)
Immunosuppression	31 (3)
Cancer	31 (3)
Other	412 (33)
Total^2^	1234
Missing	166 (15)

^1^Patients with at least one registered pre-existing disease.

^2^Total pre-existing diseases registered, some patients had more than one disease.

Characteristics of Patients Hospitalized with Coronavirus Disease 2019 in Health Centers of Southeast Network.

The mortality of the cohort was 9.77% (146 of 1495) patients. Mortality was higher for male patients 63% (92 of 146), but not significant (*p* = 0.078), also for patients aged 65 or more (OR 5.09; 95% CI 3.42/7.57), was the variable with the greatest weight in the logistic regression, and for those had some pre-existing disease (OR 2.61; 95% CI 1.63/4.19). It was unknown for 22 (15%) patients (Table 3). The hypertension, chronic lung disease, and cardiovascular disease through logistic regression showed higher risk (Table 4).

**Table 3:** Mortality, characteristics of this populations, risk factors.

Mortality No. (%) All patients 146 (9.8)		Chi Square Test (Total Patients/Mortality Patients)	Odds Ratio 95% CI (Lower/Upper)
Gender	92(Male)/51(Female)	*p* = 0.078	
Pre- existing diseases#	117 (94)	*p* < 0.001	2.61* (1.63/4.19)
Age. mean (SD)	60 (14)	*p* < 0.001	5.09** (3.42/7.57)
Safe discharge	66 (12)	*p* = 0.994	
#Severe obesity	6 (7.8)	*p* = 0.276	
#Immunosuppression	5 (20.8)	*p* = 0.157	
#Hypertension	51 (23.9)	*p* < 0.001	3.132 (2.144—4.574)
#Cancer	3 (11.5)	*p* = 0.985	
#Mellitus diabetes	33 (21.3)	*p* < 0.001	2.358 (1.532—3.628)
#Cardiovascular disease	23 (28.4)	*p* < 0.001	3.82 (2.015—5.676
#Chronic lung disease	19(22.9)	*p* < 0.001	2.438 (1.415—4.201)
#Chronic renal disease	5 (17.9)	*p* = 0.301	

*Compared deceased patients (146) vs total population with information on the history of pre-existing disease (1012).

**Odds Ratio for Age group (65 or more/up to 64).

**Table 4:** Mortality, Logistic Regression, Compared deceased patients (146) vs total population included missing cases.

Variables	Sig	Exp(B) (95 CI Lower/Upper
Hypertension	0.004	1.920 (1232/2.993
Chronic lung disease	0.020	2.019 (1.117/3.648)
Cardiovascular disease	0.009	2.128 (1.207/3.752)
Pre-existing diseases	0.019	2.072 (1.126/3.813)
Age group	0.000	3.493 (2.273/5.368)

In this group, hospitalized in ITU 73% (107) cases, 24% (35) patients in GWo and 3% (4) patients in GW. The percentage of mortality was 15% regarding the total number of discharges (976) patients. Mortality compared between populations with safe discharge with those who did not safe discharge not observed statistically difference (*p* = 0.994).

During the study period, total 3741 patients were admitted to the SN, 1495 patients (40%) with lab-confirmed COVID-19; 1262 patients (33%) were suspected COVID-19; and 984 (26%) were patients with other pathologies.

## Discussion

In this cohort investigation, NS was involved in 1495 cases hospitalized. In Argentina a total of 312,659 cases were reported in this period, no discriminated between patients hospitalized and outpatients^15^. We reviewed the indexed and non-indexed publications on hospitalized patients with a diagnosis of COVID-19 in Argentina, and we have found some works that were published after we sent our manuscript. This pulbications that present a selection of the studied population different from our work, however the results in relation to risk factors and mortality are similar to our findings^16–18^, presents an perspective different like the one carried out by us.

The health system in our region allocates 60% of the availability of beds in ITU to the pandemic, among suspected and confirmed patients, and almost 50% for General Ward. This situation certainly has a strong impact on the care of patients with other diseases who receive regular care, not yet studied this effect.

The cohort was characterized by average age of 48.9 years, similar to reported in Argentina^7^, but are younger, compared to the most publications of developed countries^19–21^ . May be due to the gap between developed and developing countries in relation to population pyramid profile, in Latin America, also observed in Argentina, those under 15 years of age represent 25.2%, and the population and those over 60 years are 15.1%^18^. While in developed countries, those over 60 reach, in some countries, double that percentage ^18^. More than half of patients were men, as well as referred most authors ^7,19^.

The cohort was characterized by substantial chronic health comorbidities, 1012 (81%) patients, these observations are consistent with most of reports every region of the world ^19–21^.

The study population included patients who live in conditions of vulnerability, we compared particularly with those who don't have housing and lived in worse conditions^22,23^. This condition of extreme vulnerability does not necessarily mean a risk factor neither more severe illness nor mortality. Could be due, to despite that still economic and social deficits of health system, major increase the number of hospital beds in the area, and optimize the resource utilization of ICUs can effectively to offer a response to our people.

The mortality of the cohort was 9.77%, was higher for patients aged 65 or more (OR 5.09), and for those had some pre-existing disease (OR 2.61). The hypertension, chronic lung disease, and cardiovascular disease through logistic regression showed higher risk. These observations are consistent with reports demonstrating older persons, and those with comorbidities have the highest risk of mortality related to COVID-19^24,25^. However, unlike other reports from developed or some developing countries, the mortality in our study is lower^20,21,24,25^. This finding may be related, to what we have already commented, the age of our cohort is younger than other published series, and the health system was able to respond to the demand. On the other hand, old individuals live at home and generally receive family support, which has allowed them to maintain social isolation^26^.

The creation of the Network to link all the health centers in the region and clinic records makes for a valuable public health investigative tool that can help to define clinical strategies for patients care during the pandemic.

## Conclusions

In this cohort involving SN response, in a developing country, urban poverty in Argentina remains (40.9% of population), while extreme poverty increased to 10.5% in 2020. In SN the the socio-economic situation is even worse, especially in the care of vulnerable patients.

The strategies that we use in our experience to face the pandemic, in particular the increase in the availability of the number of available hospital beds and human and technical resources since the beginning of the pandemic resulted essential in order to respond to the serious health situation. We consider that may be useful for other regions with similar socio-economic conditions.

## Supplementary Information

Supplementary Information.

## Data Availability

The Availability of data and materials section refers to the raw data used in your study and presenting tables and figures is not sufficient to state that all data is contained within the manuscript and additional files. Please only use this statement if you have indeed provided all raw data on which your study is based. We strongly encourage all authors to share their raw data, either by providing it in a supplementary file or depositing it in a public repository and providing the details on how to access it in this section. If you do not wish to share your data, please clearly state this in this section along with a justification. Data availability statements can take one of the following forms (or a combination of more than one if required for multiple datasets): The a part of the datasets generated and/or analysed during the current study are available in the Hospital El Cruce N Kirchner repository (https://repositorio.hospitalelcruce.org/xmlui/handle/123456789/115). The other datasets used and/or analysed during the current study available from the corresponding author on reasonable request.

## Abbreviations

AMBA: Buenos Aires Metropolitan
SN: Area Southeast Network
HEC: El Cruce Hospital N Kirchner
ITU: Therapy Unit
GWo: General Ward with oxygen therapy
GW: General Ward stay
ID: Identification of patient
