# Publisher Correction: Clinical characteristics of vulnerable populations hospitalized and diagnosed with COVID-19 in Buenos Aires, Argentina

**DOI:** 10.1038/s41598-021-96120-1

**Published:** 2021-08-27

**Authors:** A. Yacobitti, L. Otero, V. Doldan Arrubarrena, J. Arano, S. Lage, M. Silberman, M. Zubieta, I. Erbetta, P. Danei, G. Baeck, V. Vallejos, F. Cavalli, N. Calderón, M. Di Gregorio, V. Hernandez, D. Bruno, B. Rodera, I. Macherett, M. Parisi, M. Gallastegui, A. Paz, R. Bernardi, S. Azcárate, A. Hraste, I. Caridi, L. Boechi, P. Salgado, S. Kochen

**Affiliations:** 1 Network Patient Management, Hospital El Cruce N Kirchner, F. Varela, Pcia Buenos Aires Argentina; 2 Planning Area, Hospital El Cruce N Kirchner, F. Varela, Pcia Buenos Aires Argentina; 3 General Ward, Hospital El Cruce N Kirchner, F. Varela, Pcia Buenos Aires Argentina; 4 Intensive Therapy Unit, Hospital El Cruce N Kirchner, F. Varela, Pcia Buenos Aires Argentina; 5 Laboratory, Hospital El Cruce, F. Varela, Pcia Buenos Aires Argentina; 6 Administrative Area, Hospital Module N° 11, F. Varela, Pcia Buenos Aires Argentina; 7 Administrative Area, UPA N° 11, F. Varela, Pcia Buenos Aires Argentina; 8 Patient Admission, Hospital Mi Pueblo, F. Varela, Pcia Buenos Aires Argentina; 9 Prompt Attention Unit, UPA N° 5, A. Brown, Pcia Buenos Aires Argentina; 10 Administrative Area, UPA N°, 5 and Module N° 9, A. Brown, Pcia Buenos Aires Argentina; 11 Administrative Area, Hospital L. Meléndez, A. Brown, Pcia Buenos Aires Argentina; 12 Statistics, Hospital Oñativia, A. Brown, Pcia Buenos Aires Argentina; 13 Administrative Area, Hospital Oñativia, A. Brown, Pcia Buenos Aires Argentina; 14 Medical Clinic, Iriarte Hospital, Quilmes, Pcia Buenos Aires Argentina; 15 Intensive Therapy Unit, Iriarte Hospital, Quilmes, Pcia Buenos Aires Argentina; 16 Oller HospitalOller Hospital, Quilmes, Pcia Buenos Aires Argentina; 17 Administrative Area, UPA N° 17, Quilmes, Pcia Buenos Aires Argentina; 18 Intensive Therapy Unit, Evita Pueblo Hospital, Berazategui, Pcia Buenos Aires Argentina; 19 Patient Management, Evita Pueblo Hospital, Berazategui, Pcia Buenos Aires Argentina; 20 Institute of Calculation, FCEN, UBA and CONICET, Ciudad de Buenos Aires, Argentina; 21 Public Health Research Institute, University of Buenos Aires, Caba, Argentina; 22 Neurosciences and Complex Systems Unit (EnyS), CONICET‑ Hosp. El Cruce “N. Kirchner” ‑ Univ. National A. Jauretche, Fac. Med, Univ. Buenos Aires, Av Calchaqui 5401, CP B1888AAE F. Varela, Province Buenos Aires Argentina

Correction to: *Scientific Reports* 10.1038/s41598-021-87552-w, published online 06 May 2021

The original version of this Article contained an error in Affiliation 20, which was incorrectly given as “UPA N° 10, Berazategui, Pcia Buenos Aires, Argentina.” The correct affiliation is listed below:

Institute of Calculation, FCEN, UBA and CONICET, Ciudad de Buenos Aires, Argentina

The original Article has been corrected.

