# Supplementary material for: Clinical characteristics of vulnerable populations hospitalized and diagnosed with COVID-19 in Buenos Aires, Argentina
Source: Sci Rep. 2021 May 6;11:9679. doi: 10.1038/s41598-021-87552-w (PMC8102488; doi:10.1038/s41598-021-87552-w)
Supplement: Supplementary file 1 — Supplementary Information. [file 41598_2021_87552_MOESM1_ESM.docx]

**Clinical characteristics of vulnerable populations hospitalized and diagnosed with COVID-19 in Buenos Aires, Argentina**

Yacobitti A MD^1.1^, Otero L Admin^1.2^, Doldan Arruabarrena V Admin^1.2^, Arano J MD^1.3^, Lage S MD^1.4^, Silberman M MD^1.2^, Zubieta M PhD^1.5^, Erbetta I Admin^1.6^, Danei P Admin^1.7^, Baeck G MD ^1.8^, Vallejos V MD^2^, Cavalli F Admin ^2.1^, Calderón N Admin^2.2^, Di Gregorio M Admin^2.3^, Hernandez V MD^2.4^, Bruno D Admin^2.5^, Rodera B MD^3^, Macherett I MD^3.1^, Parisi M MD^3.2^, Gallastegui M MD^3.4^, Paz A Admin^3.5^, Bernardi R MD^4^, Azcárate S MD^4.1^, Hraste A MD ^4.2^, Caridi I PhD^5^, Boechi L PhD^5^, Kochen S PhD MD^1.9^

**Supplementary appendix**

**Southeast Network Public Health Institutions**

| **Municipalities** | **Hospitals^5^** | **Administrative Unit** | **Disponibility** |
| --- | --- | --- | --- |
| **Florencio Varela^1^** | **Hospital ¨El Cruce, Néstor Kirchner¨** | National and Provincial |  |
|  | **Zonal Hospital "Mi Pueblo"** | Provincial |  |
|  | **Modular Hospital N° 11** | National and Provincial |  |
|  | **¨UPA¨ N° 11** | Provincial |  |
| **Quilmes^2^** | **Zonal Hospital “Dr. I. Iriarte "** | Provincial |  |
|  | **Sub-Zonal Specialized Maternal and Children Hospital ¨Dr. Oller¨** | Municipal |  |
|  | **Modular Hospital N° 10** | National and Provincial |  |
|  | **¨UPA¨ N° 17** | Provincial |  |
| **Berazategui^3^** | **General Zonal Hospital "Evita Pueblo"** | Provincial |  |
|  | **¨UPA¨ N° 10** | Provincial |  |
| **Admiral Brown^4^** | **General Zonal Hospital “Dr. A. Oñativia "** | Provincial |  |
|  | **General Zonal Hospital “Lucio Meléndez¨** | Provincial |  |
|  | **Modular Hospital N° 9** | National and Provincial |  |
|  | **¨UPA¨ N° 5** | Provincial |  |

1. **The Municipality of Florencio Varela** is a district of the Argentine province of Buenos Aires located in the southern zone and in the second cordon of Greater Buenos Aires. It has an area of ​​190 km² and according to the projections of the (National Institute of Statistics and Censuses) it has a population of 517,082 inhabitants and its population density is 2,242 inhabitants / km².^1^

2. **The Municipality of Quilmes**, located in the southeast of Greater Buenos Aires, on the shores of the Río de la Plata, has an area of ​​125 km² and according to INDEC projections it has a population of 664,783 inhabitants and its population density is 4,663 hab / km².^1^

3. **The Berazategui Municipality** is located to the southeast of the Greater Buenos Aires conglomerate, on the coast of the Río de la Plata, it has an area of ​​217 km², a population of 365,771 inhabitants and a population density of 1,494 inhabitants/ km².^1^

4. **The Municipality of Almiral Brown**, is a party in the province of Buenos Aires that is part of the urban conglomerate, known as Greater Buenos Aires, located in the southern part of it. It has an area of ​​129 km², the number of inhabitants is 597,969 and its population density is 4286.1 inhabitants / km².^1^

5. The Health Center have different level of complexity:

The "El Cruce" Hospital, have an high complexity with University teaching.

The Zonal and Sub-zonal Hospitals, Iriarte, Mi Pueblo, Evita Pueblo, Lucio Meléndez, and Oñativia, also the Modular Hospital, recently built during the course of the current year and are located in the municipalities of Quilmes, Florencio Varela and Almirante Brown., are hospitals of medium complexity with hospitalization, according to the international classification, most of them Level 6 and 8 that have Hospitalization with Medical Clinic - Surgery - Pediatrics and Toco-gynecology with Intensive Care Services and auxiliary services for diagnosis and treatment.

The Hospital Oller, with Hospitalization of Medical Clinic - Surgery - Pediatrics and Toco-gynecology, without Intensive Unit Care.

Prompt Attention Units (UPA) are establishments of intermediate complexity with the capacity to attend emergencies, pediatric consultations, clinics, laboratory and diagnostic imaging, 24 hours a day and have hospitalization. They were built during the last 10 years, and are located in the districts of Berazategui (UPA N ° 10), Quilmes (UPA N ° 17), Almirante Brown (UPA N ° 5) and Florencio Varela (UPA N ° 11 ).

Reference

1- National Institute of Statistics and Censuses. Population by sex and five-year age groups. Years 2010-2040 [Internet]. Buenos Aires: INDEC; 2010 [cited 2020 Jul 19]. Available at: https://sitioanterior.indec.gob.ar/nivel4_default.asp?id_tema_1=2&id_tema_2=24&id_tema_3=84
